# Associations of estimated glomerular filtration rate and blood urea nitrogen with incident coronary heart disease: the Dongfeng-Tongji Cohort Study

**DOI:** 10.1038/s41598-017-09591-6

**Published:** 2017-08-30

**Authors:** Haijing Jiang, Jun Li, Kuai Yu, Handong Yang, Xinwen Min, Huanqian Chen, Tangchun Wu

**Affiliations:** 1 Department of Occupational and Environmental Health and Ministry of Education Key Lab for Environment and Health, School of Public Health, Tongji Medical College, Huazhong University of Science and Technology, Wuhan, China; 2 Department of Nutrition, Harvard T.H. Chan School of Public Health, Boston, Massachusetts USA; 3 Dongfeng Central Hospital, Dongfeng Motor Corporation and Hubei University of Medicine, Shiyan, China

## Abstract

Estimated glomerular filtration rate (eGFR) has been reported to be associated with risk of incident coronary heart disease (CHD), and blood urea nitrogen (BUN) has been shown to be a strong predictor of mortality in patients with heart failure (HF). However, such epidemiological evidence from Chinese population was still limited. We used Cox proportional-hazards regression models to investigate the associations of eGFR and BUN with risk of incident CHD in the prospective Dongfeng-Tongji (DFTJ) cohort. After fully adjusted for potential confounders, a 10-unit decline in eGFR was associated with higher risk for CHD (hazard ratio [HR] 1.05, 95% confidence interval [CI] 1.01–1.09); compared with individuals with normal eGFR levels (eGFR ≥ 90 ml/min per 1.73 m^2^), individuals with a mild-to-severe eGFR decline (15 to 60 ml/min per 1.73 m^2^) were at significantly greater risk for CHD (HR 1.25, 95% CI 1.05–1.48; *P* = 0.011). Compared with individuals in the lowest tertile of BUN, those in the highest tertile were at significantly greater risk for CHD (HR 1.17, 95% CI 1.03–1.33; *P* = 0.014). In conclusion, a mild-to-severe decline in eGFR or a raised level of BUN might be associated with increased risk of incident CHD in middle-aged and elderly Chinese populations.

## Introduction

In the past decade, chronic kidney disease (CKD), which was defined as an estimated glomerular filtration rate (eGFR) less than 60 ml/min per 1.73 m^2^ in epidemiological studies^1^, has reached an epidemic level across Asia^2^. In China, the overall prevalence of CKD was reported to be 10.8%, estimating about 119.5 million Chinese adults affected by the disease^3^. Moreover, individuals with CKD were observed to be at high risk for morbidity and mortality of a series of illnesses, especially cardiovascular disease, aggravating the burden of health care and threatening quality of life^4^. CKD needs to be paid more attention in China.

Kidney is an essential organ in the urinary system that maintains the internal homoeostasis through its complex function such as regulation of electrolytes and acid-base^5^ and maintenance of blood pressure and fluid balance^6^. The dysfunction of kidney not only implicates potential pathological changes of the kidney, but could also influence other organs and systems as a result of the violation of body homoeostasis^7^. Cardiovascular system is one of the most affected systems upon the impairment of kidney function^8–10^. Previous meta-analysis including more than 1.4 million general-population (the majority of which were Caucasians) has observed that an impaired kidney function, defined as eGFR lower than 60 mL/min per 1.73 m², was associated with an increased risk of incident coronary heart disease (CHD) independently of prevalent hypertension and diabetes^11^. Blood urea nitrogen (BUN), a metabolic product of protein that was often used to indicate kidney health^12^, has been reported to be strongly associated with hospitality and mortality among patients with acute or chronic heart failure (HF)^13–18^. However, epidemiological evidence from Chinese populations that related kidney function biomarkers (eGFR and BUN) to risk of incident CHD was still limited.

In the present study, on the basis of a prospective cohort of middle-aged and elderly Chinese populations, we investigated the associations of kidney function biomarkers (eGFR and BUN) with risk of incident CHD. We also explored the possibilities of non-linear relationships between kidney function biomarkers and CHD risk.

## Results

### Baseline characteristics

The basic characteristics of the study participants at baseline were shown in Table 1. Among the 16,989 participants qualified for analysis of eGFR, 5,261 individuals (58.8% females) with a mean baseline age of 58.5 years had normal kidney function (eGFR ≥ 90 ml/min/1.73 m^2^), 9,991 individuals (53.9% females) with a mean age of 63.0 years had mildly decreased kidney function (eGFR from 60 to 90 ml/min/1.73 m^2^), and 1,737 individuals (53.3% females) with a mean age of 67.2 years had mildly to severely decreased kidney function (eGFR from 15 to 60 ml/min/1.73 m^2^). Comparing to individuals with normal kidney function, individuals who had decreased kidney function were more likely to be males, older in age, less educated, with higher body mass index (BMI), systolic blood pressure (SBP), total triglyceride (TG), total cholesterol (TC) and fasting blood glucose levels, and more likely to have a prevalent hypertension or diabetes and a more frequent usage of diuretics.

**Table 1:** Characteristics of the study participants.

	Estimated glomerular filtration rate (eGFR)			*P*	Blood urea nitrogen (BUN)			*P*
	Group 1 (≥90 ml/min/1.73 m^2^)	Group 2 (60–90 ml/min/1.73 m^2^)	Group 3 (15–60 ml/min/1.73 m^2^)		Tertile 1 (<4.6 mmol/l)	Tertile 2 (4.6–5.7 mmol/l)	Tertile 3 (≥5.7 mmol/l)	
Participants, N (%)	5261 (31.0)	9991 (58.8)	1737 (10.2)		5316 (33.2)	5322 (33.3)	5355 (33.5)	
Female, N (%)	3091 (58.8)	5380 (53.9)	926 (53.3)	<0.001	3448 (64.9)	2968 (55.8)	2463 (46.0)	<0.001
Age (years; mean ± SD)	58.5 ± 6.4	63.0 ± 7.4	67.2 ± 7.9	<0.001	60.4 ± 7.6	62.1 ± 7.5	63.5 ± 7.6	<0.001
Education < High School, N (%)	3416 (65.4)	6553 (66.2)	1167 (67.7)	<0.001	3488 (66.2)	3464 (65.6)	3513 (66.1)	0.007
Current smoker, N (%)	1086 (20.6)	1841 (18.4)	288 (16.6)	<0.001	901 (17.0)	990 (18.6)	1132 (21.1)	<0.001
Current drinker, N (%)	1265 (23.9)	2255 (22.6)	332 (19.1)	0.002	1081 (20.3)	1206 (22.7)	1299 (24.3)	<0.001
Passive smoking, N (%)	1034 (19.7)	1860 (18.6)	295 (17.0)	0.039	1023 (19.2)	989 (18.6)	947 (17.7)	0.114
BMI (kg/m^2^; mean ± SD)	24.0 ± 3.4	24.4 ± 3.3	25.0 ± 3.4	<0.001	24.2 ± 3.4	24.4 ± 3.3	24.5 ± 3.3	0.001
Physical activity (hours/wk; median [IQR])	7.0 (3.5, 10.0)	7.0 (3.5, 10.0)	7.0 (3.5, 10.0)	0.306	7.0 (3.5, 10.0)	7.0 (3.5, 10.5)	7.0 (3.5, 10.5)	0.044
Systolic blood pressure (mmHg; median [IQR])	125 (110, 140)	130 (120, 140)	130 (120–140)	<0.001	130 (115, 140)	130 (120, 140)	130 (120, 140)	0.055
Diastolic blood pressure (mmHg; median [IQR])	80 (70, 80)	80 (70, 80)	80 (70, 85)	0.710	80 (70, 85)	80 (70, 85)	75 (70, 80)	<0.001
Total triglyceride (mmol/l; median [IQR])	1.1 (0.8, 1.6)	1.2 (0.9, 1.7)	1.4 (1.0, 2.0)	<0.001	1.21 (0.9, 1.7)	1.22 (0.9, 1.7)	1.14 (0.8, 1.6)	<0.001
Total cholesterol (mmol/l; mean ± SD)	5.1 ± 0.9	5.2 ± 0.9	5.3 ± 1.0	<0.001	5.1 ± 1.0	5.2 ± 0.9	5.2 ± 0.9	<0.001
HDL (mmol/l; mean ± SD)	1.5 ± 0.4	1.4 ± 0.4	1.4 ± 0.4	<0.001	1.4 ± 0.4	1.4 ± 0.4	1.5 ± 0.4	<0.001
LDL (mmol/l; mean ± SD)	2.9 ± 0.8	3.1 ± 0.8	3.0 ± 0.8	<0.001	3.0 ± 0.8	3.0 ± 0.8	3.0 ± 0.8	0.028
Glucose (mmol/l; median [IQR])	5.6 (5.2, 6.1)	5.6 (5.2, 7.3)	5.7 (5.2, 6.4)	<0.001	5.6 (5.2, 6.1)	5.6 (5.2, 6.2)	5.7 (5.3, 6.3)	<0.001
History of diabetes, N (%)	802 (15.2)	1723 (17.3)	402 (23.1)	<0.001	783 (14.7)	911 (17.1)	1077 (20.1)	<0.001
History of hypertension, N (%)	1256 (23.9)	3228 (32.3)	817 (47.0)	<0.001	1405 (26.4)	1658 (31.2)	1917 (35.8)	<0.001
Family history of CHD, N (%)	273 (5.2)	431 (4.3)	42 (2.4)	<0.001	258 (4.9)	248 (4.7)	185 (3.5)	0.001
Diuretics usage, N (%)	48 (1.0)	134 (1.3)	44 (2.5)	<0.001	54 (1.0)	72 (1.4)	84 (1.6)	0.041
BUN (mmol/l; median [IQR])	4.9 (4.0, 5.7)	5.1 (4.3, 6.1)	5.8 (4.8, 6.9)	<0.001	3.9 (3.5, 4.3)	5.0 (4.8, 5.3)	6.5 (6.0, 7.2)	
eGFR (ml/min/1.73 m^2^; median [IQR])	96.7 (93.1, 100.9)	77.0 (69.8, 83.3)	54.1 (49.1, 57.3)		84.9 (74.1, 94.5)	82.2 (70.7, 93.4)	77.0 (65.3, 89.4)	<0.001
Incident CHD, N (%)	380 (7.2)	1061 (10.6)	305 (17.6)	<0.001	474 (8.9)	538 (10.1)	636 (11.9)	<0.001

Continuous variables were presented as mean ± standard deviation (SD) or median (Inter Quartile Range).

Categorical variables are presented as N (%).

Differences among groups were tested using analyses of variance or nonparametric tests for continuous variables and chi-square tests for categorical variables.

We categorized the 15,993 participants qualified for analysis of BUN into three categories according to the tertiles of BUN in our study participants (Table 1). Individuals with higher BUN concentrations were more likely to be males, older in age, currently smoking, currently drinking, with higher BMI, fasting blood glucose levels and lower eGFR levels, and likewise more likely to have a prevalent hypertension or diabetes and a more frequent usage of diuretics.

### Associations of eGFR and BUN with incident CHD

Among the participants qualified for analysis of eGFR, 1,746 (10.3%) individuals developed incident CHD during a mean follow-up time of 4.5 years (standard deviation [SD] 0.6 years), while the number of incident CHD cases in analysis of BUN was 1,648 (10.3%). After basic adjustment for age, gender and examination centers, significantly higher risk of developing incident CHD was observed in individuals with mildly to severely decreased kidney function (hazard ratio [HR] 1.45, 95% confidence interval [CI] 1.23 to 1.72) comparing to individuals with normal kidney function, and each 10 units decline in eGFR was associated with increased risk of incident CHD (HR 1.09 [95% CI 1.05 to 1.13], *P*_trend_ < 0.001, Table 2). Based on the same analysis model, compared to individuals in the lowest tertile of BUN, significantly greater risk for incident CHD was observed among those in the mid and the highest tertile (HR 1.15 [95% CI 1.01 to 1.30] and HR 1.27 (95% CI 1.12 to 1.43, respectively). Each unit increment of BUN was associated with increased of risk for CHD (HR 1.07 [95% CI 1.03 to 1.10], *P*_trend_ < 0.001, Table 2).

**Table 2:** HRs (95% CI) of CHD according to groups of kidney function biomarkers.

	Groups of eGFR			*P* for trend*	HR (95% CI) per ten unit decline^#^
	Group 1 (≥90 ml/min/1.73 m^2^)	Group 2 (60–90 ml/min/1.73 m^2^)	Group 3 (15–60 ml/min/1.73 m^2^)		
**Participants**	5261	9991	1737		
**Median of eGFR**	96.7	77.0	54.1		
**Cases/Person**-**years**	380/23472.7	1061/43914.7	305/7399.4		
Age-, gender-, examination centers- adjusted	1 [Ref]	1.05 (0.93–1.19)	1.45 (1.23–1.72)	<0.001	1.09 (1.05–1.13)
Model 1	1 [Ref]	1.03 (0.90–1.16)	1.33 (1.13–1.58)	0.001	1.06 (1.03–1.10)
Model 2	1 [Ref]	1.01 (0.89–1.15)	1.25 (1.05–1.48)	0.015	1.05 (1.01–1.09)
	**Tertiles of BUN**			***P*** for trend*	**HR** (**95% CI**) **per one unit increment** ^**#**^
	**Tertile 1** (<**4**.**6** **mmol/l**)	**Tertile 2** (**4**.**6–5**.**7** **mmol/l**)	**Tertile 3** (**≥5**.**7** **mmol/l**)		
**Participants**	5316	5322	5355		
**Median of BUN**	3.9	5.0	6.5		
**Cases/Person**-**years**	474/23546.1	538/23507.0	636/23342.2		
Age-, gender-, examination centers- adjusted	1 [Ref]	1.15 (1.01–1.30)	1.27 (1.12–1.43)	<0.001	1.07 (1.03–1.10)
Model 1	1 [Ref]	1.14 (1.01–1.30)	1.27 (1.12–1.43)	<0.001	1.06 (1.03–1.10)
Model 2	1 [Ref]	1.12 (0.99–1.27)	1.20 (1.06–1.36)	0.004	1.04 (1.01–1.08)
Model 3	1 [Ref]	1.11 (0.98–1.26)	1.17 (1.03–1.33)	0.017	1.03 (1.00–1.07)

Model 1: adjusted for baseline age, gender, examination centers, smoking status, passive smoking, drinking status, Body mass index (BMI), physical activity (hours per week), total triglyceride (TG), total cholesterol (TC), high density lipoprotein (HDL), low density lipoprotein (LDL), family history of CHD.

Model 2: additionally adjusted for history of diabetes, hypertension, and history of diuretics usage.

Model 3: additionally adjusted for eGFR (only for analysis of BUN).

^*^ *P* value when we assigned the median value to each group and entered this as a continuous variable in the model and test its linear relation.

^#^HR when we use measured biomarker values as continuous variable in the model.

The associations of eGFR and BUN with incident CHD risk were kept constant after additionally adjustment for baseline BMI, smoking status, passive smoking, drinking status, physical activity (hours per week), TG, TC, high density lipoprotein (HDL), low density lipoprotein (LDL) and family history of CHD (Table 2 Model 1) and remained significant even after further adjustment for personal history of diabetes, hypertension, and diuretics usage (Table 2 Model 2) and eGFR (only for BUN analysis, Table 2 Model 3). In the fully adjusted multivariable model, significantly higher risk was observed in individuals with mildly to severely decreased kidney function (HR 1.25 [95% CI 1.05–1.48]) comparing to those with normal kidney function, and each 10 units decline in eGFR was associated with significantly increased risk for CHD (HR 1.05 [95% CI 1.01–1.09], *P*_*t*rend_ = 0.015, Table 2). Comparing to individuals whose BUN levels were in the lowest tertile, the adjusted HR for those in the highest tertile was 1.17 (95% CI 1.03 to 1.33), and each unit increment of BUN was associated with greater risk of incident CHD (HR 1.03 [95% CI 1.00 to 1.07], *P*_trend_ = 0.017, Table 2).

**Figure 1:**
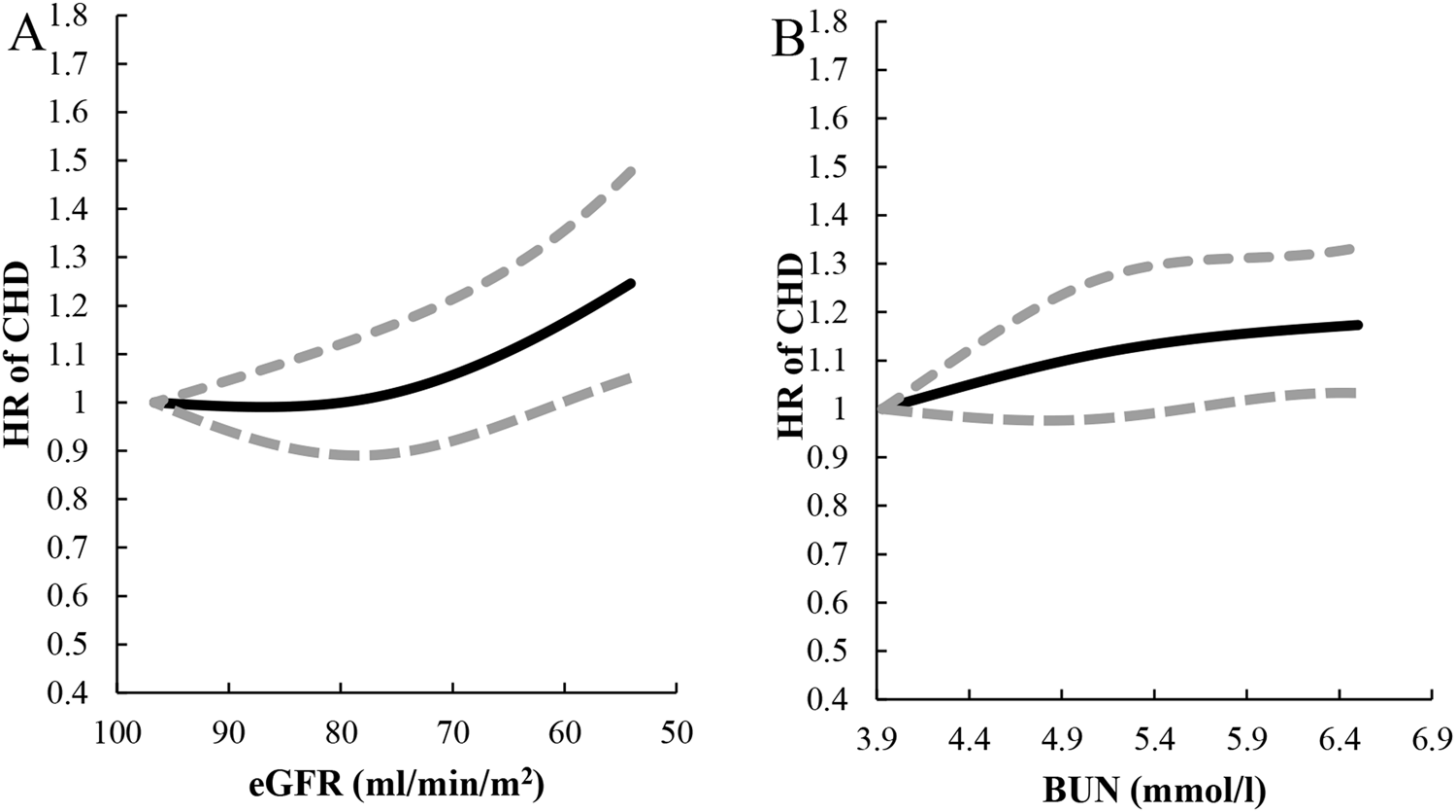
Spline plots for associations of eGFR (**A**) and BUN (**B**) with CHD risk. In each subfigure, black line represents hazard ratio (HR) according to the change of kidney function biomarkers; dotted lines depict the 95% confidence interval (CI) of HR. Covariates included in the analyses were age, gender, examination centers, smoking status, passive smoking, drinking status, Body mass index (BMI), total triglyceride (TG), total cholesterol (TC), high density lipoprotein (HDL), low density lipoprotein (LDL), family history of CHD, physical activity (hours per week), history of diabetes, hypertension, history of diuretics usage and eGFR (only for analysis of BUN).

Cubic spline curves were used to depict the relationships of eGFR and BUN with CHD risk (Fig. 1). After fully adjusted the potential covariates (the same as Model 2 for eGFR and Model 3 for BUN in Table 2), we observed an inverse association between eGFR and incident CHD risk, particularly when eGFR decreased below 60 ml/min/1.73 m^2^ (Fig. 1 subfigure A); we also observed a positive relationship between BUN levels and risk of CHD (Fig. 1 subfigure B).

### Stratified analyses

When stratified the study participants by their major characters, we observed that the association of eGFR with CHD risk were more evident among individuals who were females, younger than 60 years old, current smokers, with normal weight, without a history of diabetes, or without a history of hypertension (comparing those with mildly to severely decreased kidney function to those with normal kidney function, HRs were 1.29, 1.82, 1.53, 1.58, 1.22 and 1.36, respectively), although the interaction effects of these factors with eGFR were not statistically significant (all *P* for interaction >0.05) (Table 3). The association of BUN with CHD risk were more evident among individuals who were males, older than 60 years old, overweight, with a history of diabetes, with a history of hypertension, or had an eGFR < 60 ml/min/1.73 m^2^ (comparing those in the highest tertile to those in the lowest tertile, HRs were 1.21, 1.26, 1.28, 1.35, 1.35 and 1.96, respectively), but the interaction effects between these factors and BUN were statistically insignificant (all *P* for interaction >0.05) (Table 3).

**Table 3:** HRs (95%CI) of CHD for kidney function biomarkers stratified by relavent characteristics.

	Groups of eGFR				*P* for trend*	*P* for interaction†
	N^#^	Group 1 (≥90 ml/min/1.73 m^2^)	Group 2 (60–90 ml/min/1.73 m^2^)	Group 3 (15–60 ml/min/1.73 m^2^)		
Age						0.125
< = 60 years	6984	1 [Ref]	1.06 (0.86–1.31)	1.82 (1.26–2.62)	0.016	
>60 years	10005	1 [Ref]	0.96 (0.82–1.13)	1.15 (0.94–1.40)	0.117	
Gender						0.060
Male	7592	1 [Ref]	0.96 (0.81–1.16)	1.19 (0.92–1.53)	0.219	
Female	9397	1 [Ref]	1.05 (0.88–1.25)	1.29 (1.02–1.64)	0.033	
Current Smoking						0.609
Yes	3215	1 [Ref]	1.16 (0.90–1.50)	1.53 (1.04–2.24)	0.035	
No	13774	1 [Ref]	0.97 (0.84–1.12)	1.17 (0.97–1.42)	0.104	
Hypertension						0.287
Yes	5301	1 [Ref]	1.04 (0.85–1.27)	1.20 (0.94–1.55)	0.125	
No	11688	1 [Ref]	0.98 (0.83–1.15)	1.36 (1.08–1.73)	0.035	
Diabetes						0.593
Yes	2927	1 [Ref]	0.97 (0.74–1.26)	1.23 (0.88–1.71)	0.181	
No	14062	1 [Ref]	1.02 (0.88–1.17)	1.22 (1.00–1.50)	0.063	
BMI						0.194
<24 kg/m^2^	7930	1 [Ref]	1.20 (0.99–1.46)	1.58 (1.20–2.08)	0.001	
> = 24 kg/m^2^	8833	1 [Ref]	0.89 (0.76–1.05)	1.07 (0.86–1.33)	0.523	
	**Tertiles of BUN**				***P*** for trend*	***P*** **for interaction†**
	**N** ^**#**^	**Tertile 1** (<**4**.**6** **mmol/l**)	**Tertile 2** (**4**.**6–5**.**7** **mmol/l**)	**Tertile 3** (**≥5**.**7** **mmol/l**)		
Age						0.730
< = 60 years	6615	1 [ref]	1.00 (0.79–1.27)	0.95 (0.74–1.23)	0.706	
>60 years	9378	1 [ref]	1.16 (1.00–1.35)	1.26 (1.08–1.46)	0.004	
Gender						0.667
Male	7114	1 [ref]	1.14 (0.94–1.38)	1.21 (1.01–1.46)	0.050	
Female	8879	1 [ref]	1.09 (0.92–1.29)	1.15 (0.96–1.38)	0.123	
Current Smoking						0.364
Yes	3023	1 [ref]	1.14 (0.89–1.52)	1.29 (0.98–1.71)	0.066	
No	12970	1 [ref]	1.09 (0.94–1.25)	1.13 (0.98–1.31)	0.103	
Hypertension						0.286
Yes	4980	1 [ref]	1.24 (1.02–1.51)	1.35 (1.11–1.64)	0.003	
No	11013	1 [ref]	1.02 (0.87–1.21)	1.05 (0.89–1.25)	0.555	
Diabetes						0.703
Yes	2771	1 [ref]	1.20 (0.92–1.56)	1.35 (1.04–1.76)	0.027	
No	13222	1 [ref]	1.08 (0.94–1.25)	1.13 (0.97–1.30)	0.122	
BMI						0.579
<24 kg/m^2^	7482	1 [ref]	1.09 (0.90–1.32)	1.04 (0.85–1.27)	0.788	
> = 24 kg/m^2^	8296	1 [ref]	1.13 (0.96–1.34)	1.28 (1.08–1.51)	0.004	
eGFR						0.015
> = 60 ml/min/1.73 m^2^	14318	1 [ref]	1.08 (0.95–1.24)	1.07 (0.93–1.23)	0.393	
<60 ml/min/1.73 m^2^	1653	1 [ref]	1.49 (0.99–2.25)	1.96 (1.33–2.90)	0.001	

^*^ *P* value when we assigned the median value to each quartile and entered this as a continuous variable in the model.

In analysis of eGFR, covariates included age, gender, examination centers, smoking status, passive smoking, drinking status, Body mass index (BMI), total triglyceride (TG), total cholesterol (TC), high density lipoprotein (HDL), low density lipoprotein (LDL), family history of CHD, physical activity (hours per week), history of diabetes, hypertension, and history of diuretics usage; in analysis of BUN, covariates additionally included eGFR.

^#^Some characteristics have missing values hence not the same total N for each stratification characteristics.

^†^ *P* value for the interaction term of continuous biomarkers*categorical stratifying variable.

## Discussion

On the basis of a largely prospective cohort of middle-aged and elderly Chinese populations, we reported that a mildly-to-severely decreased kidney function was associated with a higher risk of incident CHD independently of traditional cardiovascular risk factors, in consist with previous findings based on populations of other ethnics. Moreover, we observed that an increased level of BUN was also associated with increased risk of incident CHD. Our findings added value to the current epidemiological evidence of kidney function biomarkers in relation to risk of incident CHD, suggesting that kidney function biomarkers, especially eGFR and BUN, should be considered in the risk prediction and prevention of CHD among middle-aged and elderly Chinese populations.

Several easy-to-detect serum biomarkers, i.e. creatinine, BUN and uric acid (UA), have been widely used to indicate a person’s general kidney function in clinical settings^19, 20^. Creatinine is the product of muscle creatine catabolism and urea is the primary metabolite derived from dietary protein and tissue protein turnover. Because they are filtered primarily by glomerular with little or no renal regulation or adaptation, even in the course of declining renal function, they essentially reflect rate of glomerular filtration^21^. Glomerular filtration rate (GFR) is accepted as the best overall measure of kidney function^22^. In the context of epidemiological studies, a series of algorisms were developed to use serum creatinine together with age, gender and ethnic to estimate GFR. In the present study, we chose to use the most commonly used algorithm developed by the Chronic Kidney Disease Epidemiology Collaboration (CKD-EPI)^23^, since it has been proved to be more accurate and more stable when performed in population with different age, gender and health conditions^24^, and has been used in many previous high profile publications^25^. We categorized eGFR according to the KDIGO 2012 Clinical Practice Guideline for the Evaluation and Management of Chronic Kidney Disease^26^, in which eGFR < 60 ml/min per 1.73 m^2^ was considered as chronic kidney disease (CKD).

CKD has reached an epidemic level worldwide in recent years. In 2013, the worldwide prevalence of CKD was documented to be 8–16%^27^. The social economic burden of CKD not only comes from the disease itself, but also from other expenditures on its companied diseases, including cardiovascular diseases^4^. A growing number of prospective studies have shown that, an impaired kidney function or a decreased level of eGFR was related to higher risk of all-cause and cardiovascular mortality in individuals of various ethics, including Taiwan Chinese^25, 28–31^. In epidemiological studies of Caucasian, Hispanic or African Americans, eGFR was also found to be inversely associated with risk of cardiovascular disease (CVD)^32–35^, and it was a robust and independent indicator for future risk of CVD. These data suggested that, individuals with impaired kidney function should be paid more attention in the prevention of CVD. However, although CKD prevalence was reported to be considerably high (about 10.8%) among Chinese adults^3^, current studies were mostly cross-sectional^36–38^. Prospective evidence on kidney impairment and risk of CVD in Chinese populations is still limited. The present study, for the first time, validated that eGFR were also significant predictors of incident CHD in Chinese populations using a large cohort of middle and elder aged Chinese.

Besides findings regarding eGFR, our study also shed lights on the utility of serum BUN. Urea is a waste product of the digestion of protein and is freely filtered at the glomerlus^39^. It was often used in combaniation with creatinine in clinical settings to evaluate kidney function, and was used as a powerful predicting marker for heart failure and mortality in previous epidemiological studies^14, 16, 40, 41^. Our study for the first time observed that BUN was positively in relation to risk of CHD in Chinese populations. UA is a final enzymatic product of purinemetabolism; an abnormal level of UA may hint a potential renal dysfunction or a variety of other metabolic disorders^42, 43^. UA has been reported to be positively associated with risk of incident CHD, cardiovascular disease, and death in multiple epidemiological studies including in our cohort^44–47^. These evidence together, suggested that renal dysfunction may increase the risk of CHD in Chinese populations, and serum biomarkers, namely eGFR, BUN, and UA, can provide a power risk prediction in CHD prevention.

Previous studies have proposed several potential pathophysiological mechanisms underlying the relation of eGFR and CHD risk. First, kidney disease could cause endothelial dysfunction^48^, which is recognized as one of the initial mechanisms that lead to atherosclerosis^8, 49^. Second, low-grade inflammation cause by CKD^50^ will raise oxidative stress^51, 52^, and low-grade inflammation and oxidative stress have been linked to the pathogenesis of plaque formation and plaque rupture^53^. Third, reduced kidney function itself may be a risk factor for progression of ventricular remodeling and cardiac dysfunction^35^. Moreover, many other potential mechanisms have been suggested, for instance, disordered mineral and bone metabolism may contribute to increasing cardiovascular risk in patients with CKD^54–56^. The pathophysiological mechanism underlying the relationships of eGFR and BUN with risk of CHD remains largely unknown, and more studies are warranted to investigate the mechanism as well as the clinical applications of these biomarkers.

The strengths of the present study include the following aspects. First, to our best knowledge, it is the first prospective study investigating kidney function biomarkers in relation to risk of incident CHD in a Chinese population. Our study results suggested that individuals with decreased kidney function should be paid more attention in the prevention of CHD, and that eGFR and BUN could be promising biomarkers in CHD risk prediction among Chinese populations. Second, the current study was performed on the basis of a large number of participants which increased the statistical power and limited the false positive possibility. Finally, the availability of abundant information of the study cohort enabled us to control for multiple potential confounding variables in the association analyses.

Our study has several limitations. First, the present study is an observational study based on DFTJ cohort which had only one follow-up; the possibility of residual effect, residual confounding and potential reverse causation cannot be ruled out. In addition, subjects in DFTJ cohort are mostly middle and elder aged individuals, which limited the generalization of the study conclusion. Prospective studies with longer and multiple follow-ups and clinical trials are warranted to validate our study results. Finally, eGFR is an estimated predictor based on serum creatinine and was less accurate than estimated GFR based on cystatin C. Future clinical studies with clinically measured GFR could provide more evidence. Nevertheless, eGFR is currently the most cost-efficient marker than GFR estimated with cystatin C, and thus is more practical in epidemiological situations.

In conclusion, on the basis of a prospective analysis in a Chinese middle and elder aged cohort, we found that individuals with mildly to severely decreased eGFR had a higher risk of developing incident CHD. We also observed that a higher level of BUN could be an independent risk predictor of incident CHD. Our study adds value to previous studies of kidney function and CHD events, suggesting that individuals with decreased kidney function should be paid more attention to the prevention of CHD. Future studies are warranted to validate the results and investigate the underlying mechanisms.

## Methods

### Study population

The present study was performed on the basis of DFTJ Cohort in Shiyan, Hubei, China^57^. The DFTJ cohort recruited a total of 27,009 retirees from the Dongfeng Motor Corporation (DMC) at baseline from September 2008 to June 2010. All participants went through face-to-face interviews while the special-trained investigators completed a semi-structured questionnaire accordingly. Physical and clinical information of the participants at baseline were collected through comprehensive physical examinations and clinical tests^57^. Fasting blood samples were drawn before breakfast, processed following standardized procedures, and stored under −80 °C condition. The first follow-up of the cohort was conducted in 2013, with questionnaire information followed-up by face-to-face interviews, and physical and clinical conditions re-evaluated through standard health examinations. Biological samples were also collected at the time of follow-up.

In the present study, cohort participants with a history of cancer, CVD, nephritis, end-stage renal disease (ESRD), kidney transplant or severely abnormal electrocardiogram (possible myocardial infarction [MI], atrial fibrillation, atrial flutter, pre-excitation syndrome, pacing rhythm or ventricular premature beat appeared as coupling interval) at baseline were excluded. When investigating relation of eGFR with incident CHD, after further excluding individuals without serum creatinine measurements or with potentially severe kidney failure (eGFR < 15 ml/min/1.73 m^2^), a total of 16,989 participants retained for the analysis. When analyzing association of BUN with incident CHD, after further exclusion of individuals lacking of BUN values or with potentially severe kidney failure, 15,993 participants were included in the final analysis.

This study was approved by the Medical Ethics Committee of the School of Public Health, Tongji Medical College, and Dongfeng General Hospital, DMC. All participants provided written informed consents. All the methods in the present study were carried out in accordance with the approved guidelines.

### Ascertainment of eGFR and BUN

Serum creatinine and BUN were measured in the Biochemical Laboratories of the baseline physical examination centers, using Architect Ci8200 integrated system (Abbott Laboratories. Abbott Park, Illinois, U.S.A) with Abbott diagnostic reagents following standard procedures provided by the manufacturer. eGFR was estimated based on serum creatinine concentration, gender, age and ethnic using the CKD-EPI equation^23^, i.e. eGFR = 141 × min (Scr/κ, 1)^α^ × max (Scr/κ, 1)^−1.209^ × 0.993^Age^ × 1.018 [if female]. In the formula, Scr represents serum creatinine; κ is 0.7 for females and 0.9 for males; α is −0.329 for females and −0.411 for males; min indicates the minimum of Scr/κ or 1 and max indicates the maximum of Scr/κ or 1; unit of serum creatinine should be mmol/l; unit of age should be years, and the unit of eGFR was ml/min per 1.73 m^2^. This algorithm was approved to be more accurate than the abbreviated MDRD equation^24^. The categorical variable of eGFR was defined under consideration of the KDIGO 2012 Clinical Practice Guideline for the Evaluation and Management of Chronic Kidney Disease^26^, that we categorized eGFR ≥ 90 ml/min per 1.73 m^2^ as normal kidney function, 60 and 90 ml/min per 1.73 m^2^ as mildly decreased kidney function (relative to young adults level), and 15 to 60 ml/min per 1.73 m^2^ as mildly-to-severely decreased kidney function. BUN was categorized according to its tertiles.

### Ascertainment of incident coronary heart disease

The interested outcome in the present study was incident CHD which was defined as first occurrence of non-fatal MI, fatal MI, stable angina, unstable angina, or coronary revascularization (coronary artery bypass graft or percutaneous transluminal coronary angioplasty) from baseline to December 31, 2013^58, 59^. Potential cases (reported with a diagnosis of CHD or MI) were identified through following-up interviews, and the diagnosis of CHD and its clinical subtypes was confirmed through medical records reviewed by professional clinicians. In case of the deceased cases, medical records and death certificates were both reviewed. During of mean follow-up time of 4.5 years (from baseline interview till December 31, 2013), 1,962 incident cases were identified in the DFTJ cohort subjects who were free of CVD, cancer or severely abnormal electrocardiogram. In particular, 1,746 incident cases were identified among the 16,989 subjects eligible for the analysis of eGFR, while 1,648 incident cases were identified among the 15,993 subjects eligible for the analysis of BUN.

### Assessment of baseline variables

Demographic characteristics (including age, gender, ethnicity, educational and marital status, smoking status, passive smoking, drinking status and physical activity), medical histories (including diagnosis of hypertension, diabetes, CHD, MI, stroke, cancer and nephritis) and medication usage (including aspirin, lipid lowering drugs, hypotensive drugs, antidiabetic agents, insulin, diuretics and anticoagulants) of the study participants were collected through face-to-face interviews. Height, weight, waist circumference, heart rate and blood pressures were measured by clinicians during physical examinations. Complete blood count with differential, serum liver and kidney function biomarkers, blood lipids levels (including HDL, LDL, TG, TC) and fasting blood glucose levels were measured in the biochemical laboratories of the baseline physical examination centers, following standard clinical test procedures.

Smoking status was coded as 0, 1, and 2 for never smoker, ex-smoker and current smoker. Individuals who had been smoking at least one cigarette per day for at least six months were defined as current smokers; individuals who had quitted smoking for over half a year were defined as ex-smokers; and individuals who never smoked were defined as never smokers. Drinking status was coded as 0, 1, and 2 for never drinker, ex-drinker and current drinker. Current drinkers were considered as those who had been drinking at least once per week for more than six months; ex- drinkers were considered as those who had quitted drinking for over half a year; and never drinkers were considered as those who never drank. Physical activity was defined as a dichotomous variable with 1 for physical active (regularly taking exercise with ≥20 minutes per time and ≥3 times per week for at least six months) and 0 for physical inactive. Hours of physical activity per week were calculated by multiplying average times per week by hours per time. Passive smoking was defined as inhaling tobacco smoke from surroundings. BMI was calculated as weight in kilograms divided by height in meters squared.

### Statistical analyses

eGFR was categorized into three groups according to the KDIGO 2012 Clinical Practice Guideline for the Evaluation and Management of Chronic Kidney Disease^26^, including the reference group with normal kidney function (eGFR ≥ 90 ml/min/1.73 m^2^, n = 5,261), individuals with mildly decreased kidney function (eGFR between 60 to 90 ml/min/1.73 m^2^, n = 9,991, relative to young adults levels) and individuals with mildly-to-severely decreased kidney function (eGFR between 15 to 60 ml/min/1.73 m^2^, n = 1,738). BUN was categorized according to its tertiles. Cox proportional hazards regression models were used to investigate the associations of eGFR and BUN with incident CHD. Effect estimates of higher categories of eGFR or BUN compared to the reference groups were calculated when the group variables of eGFR and BUN were included as categorical variables; *P* trends were calculated when the median values of each of the categories were included in the model as continuous variables. Effect estimates of per 10 units decline of eGFR or each unit increment of BUN were calculated when the original values of these biomarkers were included in the model. In the basic model, age, gender, and examination centers were adjusted for as covariates; in model 1, we additionally adjusted for baseline BMI, smoking status, passive smoking, drinking status, physical activity (hours per week), TG, TC, HDL, LDL and family history of CHD; in model 2, we further adjusted for history of diabetes, hypertension, diuretic drug usage; in model 3 (only for BUN analysis), we additionally adjusted for eGFR.

The nonlinear dose-response relationships of eGFR and BUN with incident CHD were investigated using restricted cubic splines^60^ and smooth curves were plotted with three knots at the 5th, 50th, and 95th percentiles of these biomarkers with maximum values set as reference for eGFR and minimum values set as reference for BUN.

We then stratified the study population according to age (≥60 or <60 years old), gender (male or female), baseline smoking status (yes or no), baseline hypertension (yes or no), baseline diabetes (yes or no), baseline BMI (≥24 or <24 kg/m^2^) and baseline eGFR levels (≥60 or < 60 ml/min/1.73 m^2^, only for BUN analysis), and calculated the associations of eGFR and BUN with incident CHD in each of the strata using Cox proportional hazards regression model with the same protocol as described above.

All statistical analyses were conducted using SAS software version 9.4 (SAS Institute, Cary, NC). All statistical tests were two-sided, and *P* values below 0.05 were considered statistically significant.
